# High-risk pooling for mitigating risk selection incentives in health insurance markets with sophisticated risk equalization: an application based on health survey information

**DOI:** 10.1186/s12913-024-10774-x

**Published:** 2024-03-04

**Authors:** A. A. Withagen-Koster, R. C. van Kleef, F. Eijkenaar

**Affiliations:** Erasmus School of Health Policy & Management, Erasmus University Rotterdam, Rotterdam, The Netherlands

**Keywords:** Health insurance, Risk equalization, Risk selection, Survey data, Risk sharing, High-risk pooling, I10-health, G22-insurance insurance companies, actuarial studies, H51-government expenditures and health

## Abstract

**Background:**

Despite sophisticated risk equalization, insurers in regulated health insurance markets still face incentives to attract healthy people and avoid the chronically ill because of predictable differences in profitability between these groups. The traditional approach to mitigate such incentives for risk selection is to improve the risk-equalization model by adding or refining risk adjusters. However, not all potential risk adjusters are appropriate. One example are risk adjusters based on health survey information. Despite its predictiveness of future healthcare spending, such information is generally considered inappropriate for risk equalization, due to feasibility challenges and a potential lack of representativeness.

**Methods:**

We study the effects of high-risk pooling (HRP) as a strategy for mitigating risk selection incentives in the presence of sophisticated– though imperfect– risk equalization. We simulate a HRP modality in which insurers can ex-ante assign predictably unprofitable individuals to a ‘high risk pool’ using information from a health survey. We evaluate the effect of five alternative pool sizes based on predicted residual spending post risk equalization on insurers’ incentives for risk selection and cost control, and compare this to the situation without HRP.

**Results:**

The results show that HRP based on health survey information can substantially reduce risk selection incentives. For example, eliminating the undercompensation for the top-1% with the highest predicted residual spending reduces selection incentives against the total group with a chronic disease (60% of the population) by approximately 25%. Overall, the selection incentives gradually decrease with a larger pool size. The largest marginal reduction is found moving from no high-risk pool to HRP for the top 1% individuals with the highest predicted residual spending.

**Conclusion:**

Our main conclusion is that HRP has the potential to considerably reduce remaining risk selection incentives at the expense of a relatively small reduction of incentives for cost control. The extent to which this can be achieved, however, depends on the design of the high-risk pool.

**Supplementary Information:**

The online version contains supplementary material available at 10.1186/s12913-024-10774-x.

## Background

Many social health insurance markets are organized according to principles of regulated competition [1, 2]. In such markets, the government enforces regulations to safeguard affordability and accessibility of insurance coverage, while competition among health insurers aims to ensure efficiency of insurance products and good quality care. To achieve affordability of basic coverage, these markets typically include premium-rate restrictions. However, a downside of such restrictions is that they create predictable profits on healthy individuals and predictable losses on the chronically ill. These predictable profits and losses confront health insurers with incentives for risk selection, which is undesirable as risk selection might violate fairness and efficiency [3, 4, 5]. Therefore, another typical feature of regulated competitive health insurance markets is risk equalization. Risk equalization compensates insurers for predictable spending variation across individuals and thereby mitigates selection incentives. However, risk-equalization formulae currently used in practice do not (yet) account for all predictable spending variation, implying that some selection incentives remain [4, 6, 7, 8, 9].

The risk-equalization model applied in the Dutch health insurance system is considered to be one of the most sophisticated in the world. This model includes an extensive set of demographic, socioeconomic and morbidity-based risk adjusters. Nevertheless, it has been shown that even this model leaves considerable predictable profits and losses on selective groups. Using information from a large health survey, Van Kleef et al. [4] found a predictable profit of around 180 euros per person per year for consumers who reported a (very) good health status in the prior year (about 75% of the population) and a predictable loss of around 500 euros per person per year for consumers who reported a fair or (very) poor health status in the prior year (around 25% of the population). In addition, the authors found predictable losses at the level of specific chronic conditions. For example, for individuals who reported to have ever suffered from diabetes, stroke, heart attack or cancer, they found predictable losses of around 130, 900, 380 and 430 euros per person per year, respectively. These predictable profits and losses might lead insurers to engage in risk selection, e.g., via the design and marketing of insurance plans. Recently, there have been strong signals that health insurers in the Netherlands indeed engage in risk selection [10, 11, 12]. The predictable profits and losses on the abovementioned subgroups suggest that health survey information is predictive of ‘residual spending after risk equalization’ (i.e., actual spending minus predicted spending generated by the risk-equalization model). This implies that, in theory, health survey information could be used for improving the predictive power of risk-equalization models [13, 9]. However, this information is currently not used in risk-equalization models due to feasibility challenges and a potential lack of representativeness (e.g., due to selective non-response). In general, it will typically be considered too costly and practically infeasible to routinely collect this information for the entire population [13, 14]. Health insurers, however, are not restricted by these requirements. They may use information from self-administered health surveys for their own risk assessments and in doing so, find indications of profits or losses for selective groups, creating incentives for risk selection. In addition to health survey information, a health insurer might have other data that they can use for their own risk assessments. In this study, however, we focus on health survey data.

A promising but understudied option to exploit the predictiveness of survey information for residual spending after risk equalization is high-risk pooling (HRP). This is a form of risk sharing between health insurers and the regulator that can be useful in mitigating remaining selection incentives [15]. With HRP, health insurers can assign certain enrollees with high expected (residual) spending to a pool before the start of a contract period using, for instance, health survey information. For enrollees in the pool, the insurer receives a compensation based on the actual (residual) spending of these enrollees once the contract period has ended [16].

While HRP can reduce selection incentives, it also comes with a price: like other forms of risk sharing, HRP reduces incentives for insurers to control costs because the compensation they receive becomes partly dependent on actual spending [17, 18, 15]. When it comes to the design of HRP, a challenge is to maximize the reduction in selection incentives given a certain loss in incentives for cost control. This can be done by targeting risk sharing payments to where they are most needed, i.e., by assigning those enrollees to the risk pool for which insurers face the largest predicted losses.

The aim of this paper is to study the extent to which HRP based on health survey information can mitigate the risk selection incentives that remain after sophisticated risk equalization. In addition, for various HRP modalities, we assess the tradeoff between incentives for risk selection and incentives for cost control. To achieve these objectives, we simulate predictable profits and losses for selective groups under the sophisticated Dutch risk-equalization model of 2016 supplemented with HRP. We use administrative data (*N* ≈ 16.9 m) on both actual and predicted spending of 2013, merged with health survey data from 2012 (*N* ≈ 384k). To assess the effect of design choices on incentives for selection and cost control, we examine different pool sizes and methods for identification of pool members.

This paper builds on earlier work by Van Barneveld [16, 17, 19] who was the first to study HRP as a supplement to risk equalization. Our study is the first to investigate health survey information to prospectively assign enrollees to a high-risk pool in the context of sophisticated risk equalization. The novelty of this study is therefore not to be found in the HRP itself, but in the use of this approach to exploit the predictiveness of health survey information in health plan payment systems.

This paper is structured as follows. Sect. 2 presents an overview of design options for HRP. Section 3 describes the setting of the study, as well as the data and methods used. Next, Sect. 4 presents the results and Sect. 5 discusses the main findings.

## High-risk pooling design choices

Risk equalization can be supplemented with risk sharing to protect insurers against large losses and to mitigate risk selection incentives. However, risk sharing can also reduce incentives for cost control because payments to health insurers become (partly) dependent on actual spending. The extent to which incentives for cost control and risk selection are mitigated depends on the specific design of the risk sharing scheme. For HRP, there are three key design choices: Who is assigned to the pool and by whom? Which and how much of the spending included in HRP is compensated for? And how are these compensations financed [19]?. We discuss these choices in more detail below.

### Assignment of members to the high-risk pool

The first design choice is on the procedure for assigning individuals to the high-risk pool and the size of the pool in terms of the number of individuals included. HRP is based on prospective assignment of individuals using information that is available at the start of the contract period [19, 15]. In theory, the assignment of members to the pool can either be done by the regulator or by health insurers themselves. However, when the regulator wants to exploit the predictiveness of (for instance) survey information for residual spending after risk equalization, assignment will most likely be done by health insurers since they typically possess (or are in a better position to obtain) this type of information. A relevant question is then which enrollees health insurers should assign to the pool. Ideally, these are the enrollees with the highest predicted losses.

Regarding the size of the pool, the regulator can decide to use a fixed size for all health insurers or let it vary among insurers depending on differences in the risk profile of the insured population of the insurers [16, 17].

### Compensation for spending in the pool

The second design choice concerns the compensation for the spending of the individuals in the pool. The regulator has many options in this regard^1^. For instance, it can decide to compensate insurers for all spending of pooled members, for spending above a certain threshold, or for a certain percentage of spending (above a threshold) [19]. Another option is to compensate insurers on the basis of *residual* spending instead of *actual* spending. The main advantage of this is that the compensation is allocated to those individuals in the pool that have the highest residual spending after risk equalization (i.e., those with the highest losses), and not for individuals in the pool whose actual spending turns out to be well-compensated for by the risk-equalization model itself [20].

### Financing of the pool

The final design choice concerns the financing of the high-risk pool. This can be done externally or internally. With external financing, there is an external flow of money towards the payment system. In the case of internal financing, the high-risk pool is financed by a mandatory contribution from all health insurers. This contribution can be calculated at the end of the contract period or at the start, for example in the form of a proportional or flat reduction of the individual-level risk-equalization payments [19]. Alternatively, the high-risk pool can be financed by a “repayment” of profits for enrollees who are not assigned to the high-risk pool and– in retrospect– turn out to be heavily overpaid by the risk equalization model. This idea was recently proposed by McGuire et al. [21] who have shown that insurers can be heavily overpaid for relatively low-cost enrollees with one or more morbidity flags. Although we do not investigate this option in this paper, this could be an interesting direction for future research.

## Study setting, data and methods

### Study setting

This study was performed in the context of the Dutch basic health insurance, which covers physician services, hospital care and prescription drugs, among other care services. The basic health insurance operates according to principles of regulated competition. This means that health insurers compete on price and quality of insurance plans, while the regulator enforces regulation to protect public objectives such as accessibility and affordability of basic coverage. These regulations include a standardized benefits package, open enrollment, community rating per insurance plan, an individual mandate to buy health insurance, and risk equalization [22].

This paper focuses on the Dutch risk-equalization model of 2016. From 2016 to 2022 this model has undergone only relatively minor changes. The Dutch risk-equalization scheme consists of three separate models: one for somatic care, one for mental care and one for out-of-pocket spending under the mandatory deductible (385 euros in 2022). This study focuses on the model for somatic care (comprising approximately 90% of total spending under the basic health insurance), which contains a broad set of demographic, socioeconomic and morbidity-based risk adjusters. The model of 2016 contains the following risk adjusters: age interacted with gender, region, socioeconomic status and source of income both interacted with age, Pharmacy-based Cost Groups (PCGs), Diagnosis-based Cost Groups (DCGs), Multiple year High Cost Groups (MHCGs), Durable Medical Equipment Cost Groups (DMECGs), yes/no morbidity^2^ interacted with age, physiotherapy-spending in the prior year, geriatric rehabilitation care spending in the prior year and home care spending in the prior year [22]. The risk-equalization payments under the somatic model are completely prospective and not supplemented with any risk sharing payments.

### Data

To examine the effect of HRP and specific design choices on incentives for cost control and risk selection, we used two data sources. The first dataset contains administrative information on individual-level spending and risk adjusters for all Dutch citizens with a basic health insurance in 2013 (*N* ≈ 16.9 million). These data are those that were actually used to estimate the Dutch risk-equalization model of 2016.

We merged these data with health survey data from 2012 (*N* ≈ 384k) using an anonymized individual-level identification key. The health survey data contain indicators of self-reported health and lifestyle for individuals who were 19 years or older on September 1, 2012 [23]. Specifically, these data include information on self-reported general health, nineteen chronic conditions that individuals could report to have suffered from in the last 12 months and four conditions they could report to have ever suffered from. We used these data for two purposes: (1) to predict residual spending from the perspective of a health insurer (see Sect. 3.3.1 for details) and (2) to evaluate predictable profits and losses for subgroups that are potential targets of risk selection by health insurers. Our selection of groups has been extensively analyzed in previous studies and is considered relevant when it comes to the evaluation of the Dutch risk-equalization model in terms of selection incentives [4, 24, 25, 26].

Before conducting the analyses, we improved the representativeness of the health survey sample through a raking procedure. Via an iterative process we generated a weight for every record in our data using a set of key variables present in both the total adult population and the health survey. Application of these weights to the survey sample makes sure that the frequencies of these key variables in the health survey equal those in the population [27, 28]. The set of key variables includes all risk-adjuster classes of the Dutch risk-equalization model of 2016, as well as 18 quantiles of mean curative somatic spending and a proxy for whether someone had died in 2013.^3^ More details on the raking procedure and results on the sample’s representativeness before and after raking are provided by Withagen-Koster et al. [26].

### Methods

Our analytical approach to evaluate the extent to which HRP can mitigate risk selection incentives and at what cost, comprised three steps. We first simulated the assignment of individuals to the pool by insurers based on health survey information. To do so, we developed a model that uses the health survey information to predict individual-level residual spending after risk equalization. Based on the predictions from this model, we assigned the top X% with the highest predicted residual spending to the pool. For the individuals in the pool, 100% of actual spending above a certain threshold is compensated. Second, we calculated the mean profits and losses after risk equalization for selective groups identified in the health survey, with and without HRP. Lastly, using summary measures we quantified the effect of our HRP-modalities on incentives for risk selection and cost control.

The following sections describe our approach in more detail. Sect. 3.3.1 and 3.3.2 start with answering the design questions raised in Sect. 2 for our specific application of HRP. Next, Sect. 3.3.3 explains how we evaluated the effect of HRP on the predictable profits/losses for selective groups. Lastly, Sect. 3.3.4 describes how we assessed the effect of HRP on incentives for risk selection and cost control.

#### Compensation of the high-risk pool and assignment of enrollees to the pool

In this study, spending of pool members will be compensated for on the basis of residual spending above a certain threshold. This threshold was chosen such that the mean residual spending for the group of people assigned to the high-risk pool equals zero. An algorithm was used to determine the exact value of the threshold, which we determined separately for each pool size. We included 5 different pool sizes: the top-1%, top-2%, top-3%, top-4% and the top-5% of predicted residual spending.

We assumed that assignment of individuals to the pool is done by health insurers before the start of a contract period, using health survey information. Since the compensation is based on residual spending, health insurers will want to identify and assign individuals with the highest expected residual spending to the pool. A key question is how an insurer could determine the expected residual spending. First, the relationship between health survey information and residual spending must be determined, which can be done by developing a prediction model using data from a prior period. Using individual-level residual spending^4^ and health survey information from a prior period, a health insurer can develop a model to predict individual-level residual spending for the upcoming contract period. This model can be developed with conventional parametric regression methods (like ordinary least squares; OLS), which are mostly used in risk equalization, but also with statistical methods that have been developed more recently, like machine learning. Prior work has shown that machine learning algorithms have the potential to better exploit predictive information [29] and might be better in classifying individuals with the highest predicted loss. In this study we explored both options. Specifically, we used an OLS regression with stepwise selection, as well as a random forest (RF) procedure to predict residual spending using information from the health survey. Other studies have used RF in the context of risk equalization and found that RF performed well in predicting healthcare spending [29, 30]. The RF procedure creates an ensemble of many individual decision trees to protect from outliers and overfitting, with the final model being based on the average values of all estimated trees [29]. In our application, we ran 100 trees^5^ with a minimum sample size of 100 in each end node.

We randomly selected 70% of the survey sample and trained the OLS and the RF model on this section of the data (called the ‘training sample’). To evaluate model performance, we tested both models on the remaining 30% of the data (called the ‘test sample). Both models yielded a prediction of the residual spending of individuals in the training and test sample. In the Results section, a comparison of model fit between the training and test sample will reveal that “overfitting” is more of an issue with RF than with the OLS.

#### Financing of the pool

The high-risk pools in our analyses are financed by a reduction of the risk-equalization payment for individuals not assigned to the pool (i.e., the complementary group). In line with prior research, we chose a retrospective flat-rate contribution to ensure that the contribution is independent of the risk of enrollees [17]. We calculated this contribution as the total amount of money needed to fund the pool (i.e., the sum of actual residual spending of all individuals in the pool) divided by the number of enrollees in the complementary group. We then subtracted this fixed amount from the risk-equalization payment of every individual in the complementary group.

#### Predictable profits and losses for selective groups

We evaluated the effect of HRP on the incentives for risk selection and cost control using the test sample only. We did this to simulate how the implementation of HRP would likely occur in practice. That is, an insurer would train his prediction model on data from a prior contract period and apply it to a new contract period.

To evaluate the effect of HRP on the incentives for risk selection, we determined the predictable profits and losses for selective groups that are potential targets for risk selection, in two steps. First, we determined the groups for evaluation based on the health survey information. We selected nineteen groups with specific chronic conditions individuals could report to have suffered from in the past 12 months and four groups with chronic conditions individuals could report to have ever suffered from in the past. In addition, we evaluated the mean per person profits/losses for the two groups yes/no chronic condition (constructed based on the 23 groups mentioned above) and two groups based on self-reported general health: the group who reported to have a fair or (very) poor general health and the groups who reported to have a (very) good general health. As a next step– separately for each of five high-risk pool sizes studied in this paper - we determined the mean per person profit/loss for these groups by subtracting the actual spending from the revenue (i.e., spending predicted by the risk-equalization model plus the HRP contribution minus financing).^6^

#### Incentives for cost control versus incentives for risk selection

Implementing risk sharing like HRP comes with a trade-off between incentives for cost control and incentives for risk selection. To quantify the impact of HRP on incentives for cost control, we performed a simulation of ‘power’ in the spirit of (though not exactly the same as) Geruso and McGuire [31]. The ‘power’ of a payment system depends on the link between marginal costs and marginal revenues. In general, power yields a value between 0 and 1, with a value closer to 1 indicating stronger incentives for cost control, ceteris paribus. A value of 1 indicates that revenues are completely independent of costs, which will be true for a risk equalization model based on age and gender only. A value of 0 indicates that revenues are fully dependent on costs, which will be true for a payment system with 100% cost-based compensations. Combinations of risk equalization and risk sharing are found to be somewhere in between these extremes. In our analyses, we simulated the impact of HRP on power by calculating the extra revenue a health insurer receives as a result of a 10% increase in spending^7^, ceteris paribus^8^. More specifically, we define the reduction in power as the “total change in revenues” (in euros) divided by “the total change in spending” (in euros), when spending increases by 10%. We do this for each of the high-risk pool sizes.

To assess the incentives for risk selection, we calculated the weighted mean absolute result (WMAR). We first determined the mean result for each subgroup:

$$ {\stackrel{-}{e}}_{g}={\stackrel{-}{Y}}_{g}-{R}_{g}$$

Where $$ {\stackrel{-}{e}}_{g}$$ is the mean residual spending of subgroup g, $$ {\stackrel{-}{Y}}_{g}$$ is the mean actual spending of subgroup g and $$ {R}_{g}$$ is the revenue for subgroup g. The WMAR is then calculated as follows:

$$ WMAR=\frac{{\sum }_{g}\left({N}_{g}*\left|{\stackrel{-}{e}}_{g}\right|\right)}{{\sum }_{g}{N}_{g}}$$

Where N_g_ is the number of individuals in subgroup g and $\left|{\stackrel{-}{e}}_{g}\right|$ is the absolute mean residual spending of subgroup g. A higher WMAR indicates stronger incentives for risk selection. The WMAR can be based on any selection of subgroups. Ideally, this selection includes those subgroups that are potential targets of risk selection. It is not clear, however, whether selection by insurers is likely to occur at the more general level of yes/no chronically ill or at the more specific level of particular chronic diseases. Instead of making an assumption about the level on which selection takes place, we calculated the WMAR for four different sets of groups: (1) The group who reported not to suffer from a chronic condition and the groups of individuals who reported to have ever suffered from diabetes, stroke, heart attack or cancer, (2) all subgroups analyzed in this paper, (3) yes/no at least one chronic condition and (4) self-reported general health.

## Results

This section starts with a presentation of some descriptive statistics of the survey sample (Sect. 4.1), followed by results from the OLS and RF prediction models (Sect. 4.2). As we will show, the RF model performs somewhat better in identifying unprofitable individuals (i.e., those with high residual spending post risk equalization) and will therefore be used in the rest of our analysis. Based on the RF model we define five sizes of the high-risk pool. After describing these pools (Sect. 4.3), we present the mean per person profit/loss for selective subgroups of interest under each pool size (Sect. 4.4). Lastly, we provide insight in the tradeoff between risk selection incentives and incentives for cost control (Sect. 4.5).

### Descriptive statistics

Table 1 shows descriptive statistics of the rebalanced survey sample and the in terms of ‘age’ corresponding population in the administrative data. The relative frequencies of the survey sample and the population match very well. The last two columns show the relative frequencies of the samples used to train and test the prediction models. The mean spending of the training and test sample are slightly different from that of the total survey sample and population, but the relative frequencies for the age categories and for the morbidity-based risk adjusters correspond relatively well.

**Table 1:** Mean curative somatic spending and population frequencies in 2013 for selected risk-adjuster variables in the rebalanced survey sample, training sample and test sample, and the population (19 years or older on September 1, 2012)

	Survey sample (rebalanced)	Population	Training sample	Test sample
N	384,004	12,774,886	268,533	115,473
Mean spending in euros	2460	2460	2465	2448
Man, 19–34 year	11.8%	11.8%	11.7%	12.1%
Man, 35–44 year	8.7%	8.7%	8.7%	8.7%
Man, 45–54 year	9.8%	9.8%	9.7%	10.1%
Man, 55–64 year	8.4%	8.4%	8.5%	8.4%
Man, 65 year and older	10.1%	10.1%	10.1%	10.1%
Woman, 19–34 year	11.8%	11.8%	11.8%	11.6%
Woman, 35–44 year	8.8%	8.8%	8.9%	8.6%
Woman, 45–54 year	9.8%	9.8%	9.8%	9.8%
Woman, 55–64 year	8.4%	8.4%	8.5%	8.4%
Woman, 65 year and older	12.4%	12.4%	12.4%	12.3%
PCGs > 0	24.1%	24.1%	24.2%	24.7%
DCGs > 0	11.5%	11.5%	11.5%	11.7%
MHCGs > 0	7.1%	7.1%	7.0%	7.2%
DMECGs > 0	1.1%	1.1%	1.1%	1.0%
Physiotherapy spending in the previous year	2.6%	2.6%	2.6%	2.5%
Home care spending in the previous year	2.6%	2.6%	2.6%	2.6%
Geriatric rehabilitation care spending in the previous year	0.3%	0.3%	0.3%	0.2%

*Note: PCGs are Pharmacy-based Cost Groups, DCGs are Diagnosis-based Cost Groups, MHCGs are Multiple year High-Cost Groups and DMECGs are Durable Medical Equipment Cost Groups*

### Assigning members to the high-risk pool: OLS versus RF

In our simulation, we assume that health insurers would assign individuals with the highest expected residual spending to the high-risk pool. A crucial question then is how insurers could determine the expected residual spending of their enrollees, assuming they have ‘external’ information that is not already included in the risk-equalization model (in this paper: health survey data) that they can use to predict the residual spending of their enrollees. In terms of prediction models, insurers have a universe of options. We explored two of those options: an OLS regression and a RF model. Our results show that both methods yield different results in terms of which individuals are selected for the top X% of predicted residual spending.

**Fig. 1:**
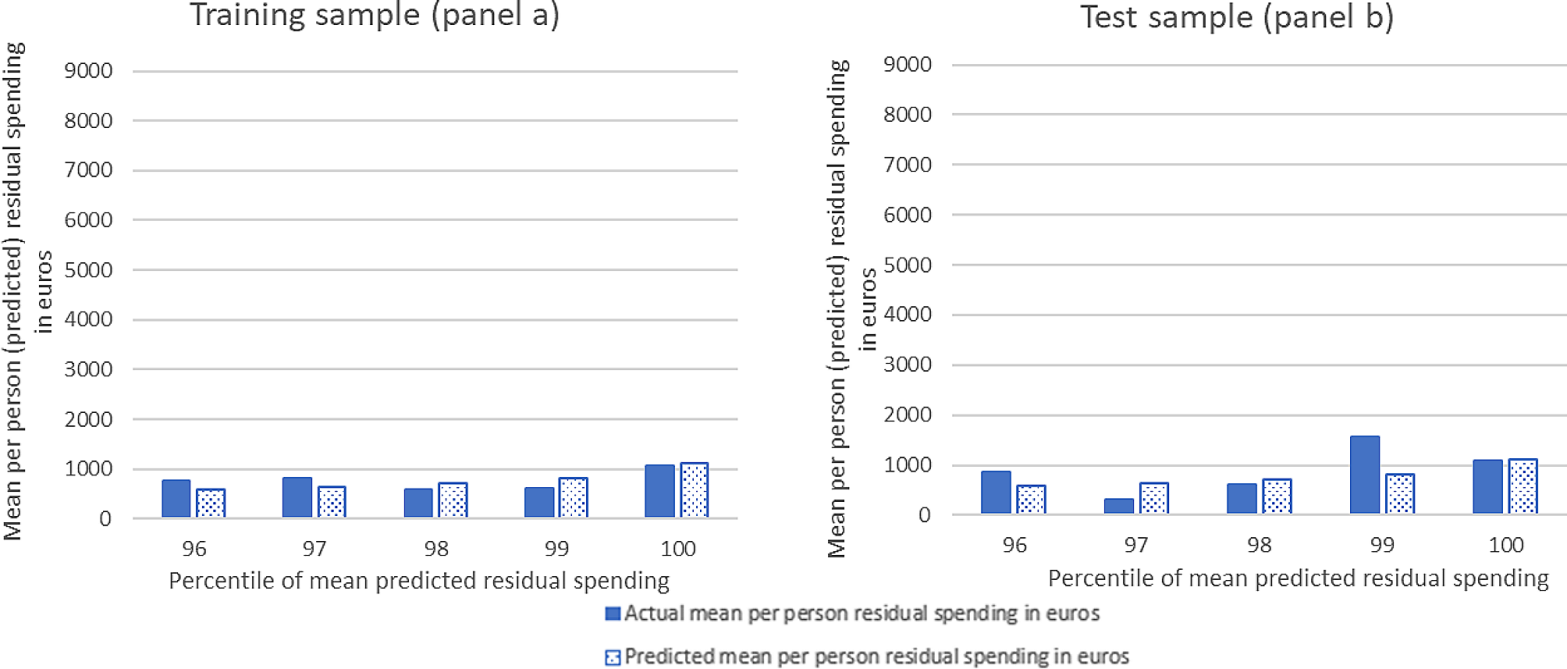
Mean per person (predicted) residual spending for the highest percentiles of predicted residual spending of the OLS prediction model for the training and test sample

Figure 1 shows the mean actual and mean predicted residual spending for the five top percentiles of predicted residual spending for the training (panel a) and test (panel b) sample for the OLS regression. The predicted residual spending (scattered bars) and the actual residual spending (filled bars) match relatively well for the training sample. For the test sample, the predicted and actual residual spending match less well than for the training sample. This was to be expected since the model was calibrated on the training sample.

**Fig. 2:**
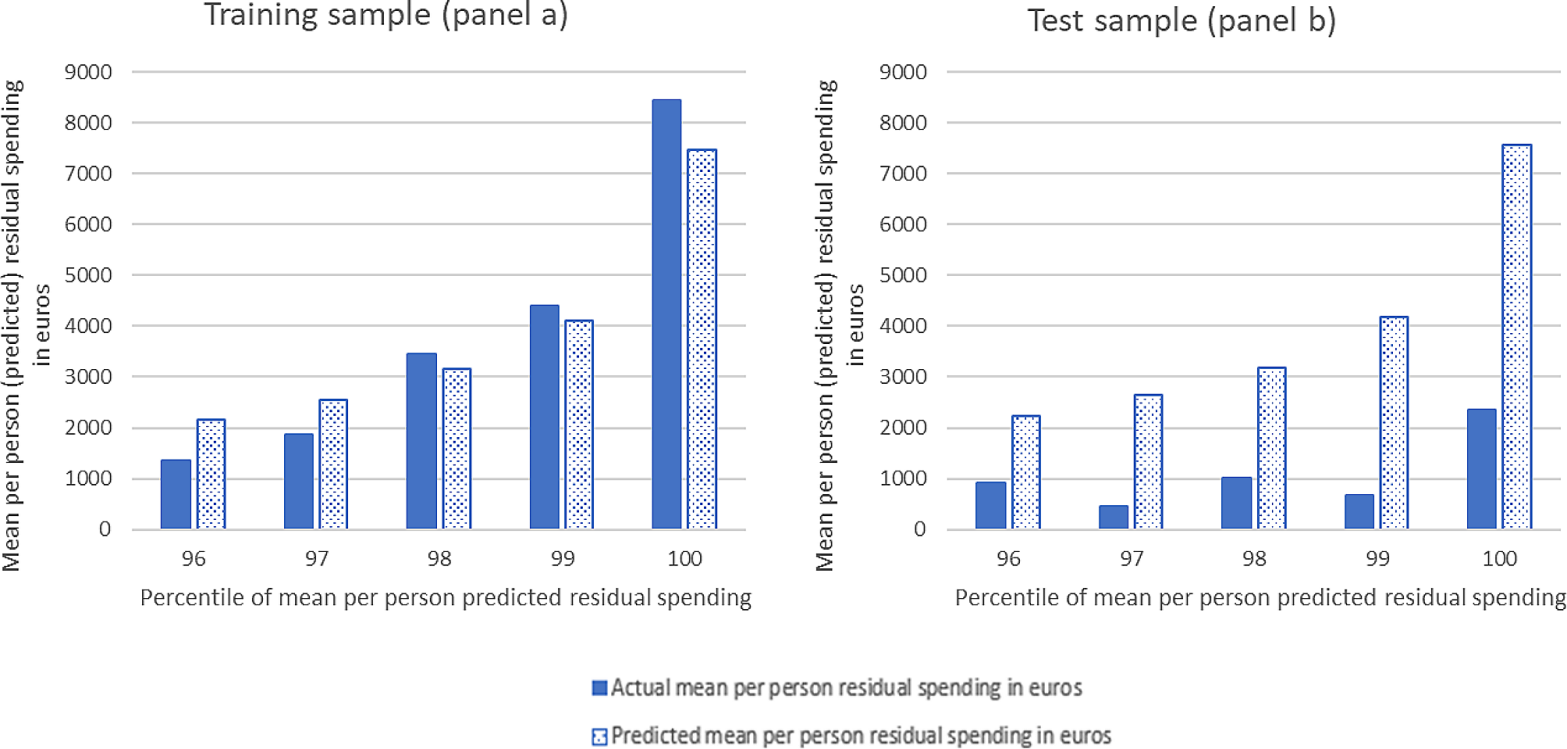
Mean per person (predicted) residual spending for the highest percentiles of predicted residual spending of the random forest model for the training and test sample

Figure 2 shows the same information as Fig. 1 but then for the RF model. The predicted and actual residual spending match relatively well for the training sample (panel a) and the actual residual spending (filled bars) shows an upward trend. For the test sample (panel b), predicted and actual residual spending diverge which illustrates the problem of overfitting in the training sample. In the remainder of our analysis, we will therefore use the test sample to calculate and evaluate outcomes.

When it comes to the assignment of individuals to the high-risk pool, the top X% of predicted residual spending identified by RF is more selective than that identified by OLS, which means RF is better able to identify individuals with high residual spending. This is illustrated in Table 2, which shows the mean per person actual residual spending for the top-1% to top-5% of predicted residual spending. Based on these findings, we decided to continue our analyses with only the top X% groups as identified by the RF model.

**Table 2:** Mean per person actual residual spending in euros for the top X% of predicted residual spending under the OLS and random forest prediction model

	Top 1%	Top 2%	Top 3%	Top 4%	Top 5%
OLS	1091*	1331*	1093*	895*	886*
Random forest	2359*	1506*	1343*	1130*	1086*

*Note: Results are based on test sample (* *N* = 115,473). An asterisk (*) means that the presented value is statistically significantly different from zero ( *p* *< 0.05)*

### Descriptive statistics of the five high-risk pools

Table 3 shows descriptive statistics for each of the five high-risk pools (i.e., top-1% to top-5% of predicted residual spending based on the RF model). The table shows that with an increase of the pool size, both mean spending and mean predicted spending decrease. This could be expected as the groups become less selective when more individuals with lower (residual) spending are included (see also Table 2). The threshold reflects the value above which residual spending for those in the pool is reimbursed, which is chosen such that the mean residual spending of the pool becomes zero. The threshold increases with pool size because the pool becomes less selective and therefore the mean residual spending for the group included in the high-risk pool decreases (see Table 2). This means that– on average per person in the pool– less money is needed to reduce the mean residual spending of the pool to zero. However, more individuals are included in the pool which means that the *total* costs of financing HRP increases.

**Table 3:** Mean (predicted) spending in euros under risk equalization without a high-risk pool, the threshold for compensation and the percentage of total costs needed to finance the high-risk pool for five different pool sizes (i.e., top-1% to top-5% of predicted residual spending)

	High-risk pools based on predicted residual spending from the random forest model				
	Top 1%	Top 2%	Top 3%	Top 4%	Top 5%
Mean spending	12,730	10,826	10,097	9571	9280
Mean spending predicted by the risk-equalization model	10,371	9320	8755	8440	8194
Threshold	12,961	18,066	20,408	23,706	23,807
HRP financing costs as percentage of total spending in test sample	0.96%	1.2%	1.6%	1.8%	2.2%

*Note: all spending is in euros. Results are based on test sample (* *N* *= 115,473)*

### Mean per person profits and losses for selective subgroups under high-risk pooling

**Fig. 3:**
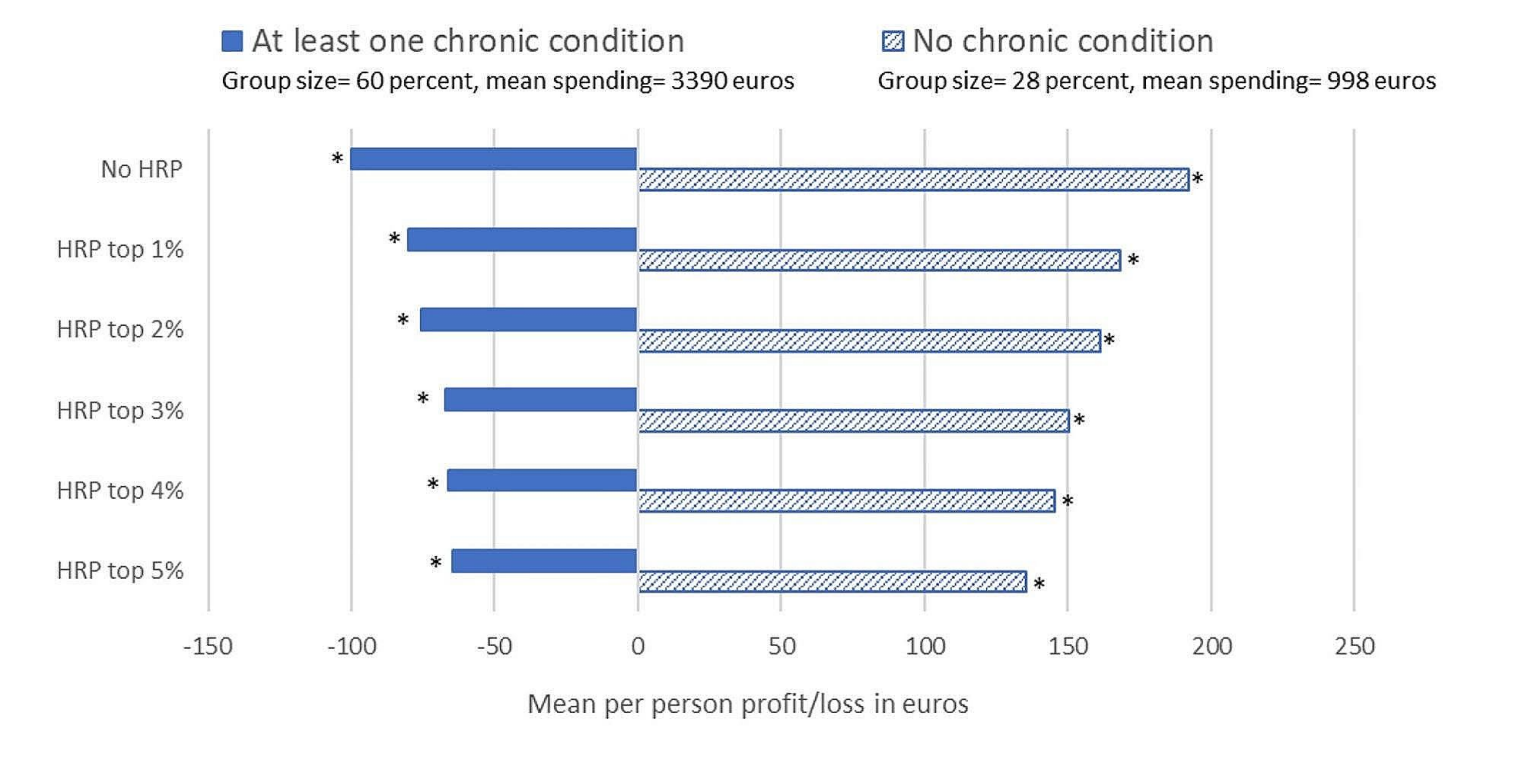
Mean per person profit/loss per year in euros under the Dutch risk-equalization model of 2016 without a high-risk pool and for 5 high-risk pools that vary in size for the groups of individuals who reported (not) to suffer from a chronic condition (ever or in the past 12 months). *Note*: Results are based on test sample (*N* = 115,473). HRP stands for high-risk pooling. No HRP shows the mean per person profit/loss under the risk-equalization model without a high-risk pool. An asterisk (*) means that the presented value is statistically significantly different from zero (*p* < 0.05). Group size does not sum to 100% over the two groups due to missing values for these groups

This section presents the effects of HRP on the mean per person profits/losses for selective subgroups identified in the survey sample. Figure 3 shows the mean profit/loss under the Dutch risk-equalization model with and without HRP for the individuals who reported to have suffered from at least one chronic condition (ever or in the past 12 months) and for the complementary group of individuals who reported not to have suffered from a chronic condition. As expected, the loss for the group with at least one chronic condition decreases with the pool size. The profit for the group without a chronic condition also decreases because of the zero-sum nature of the risk-equalization model, which means that over two complementary groups the mean profit/loss equals zero^9^.

Figure 3 further shows that the absolute difference in compensation between the group with and the group without a chronic condition decreases from 292 euros for the situation without HRP to 200 euros for the situation with HRP for the top 5% of predicted residual spending, a reduction of 32%. The risk pool for the top 1% is responsible for roughly half of this reduction.

**Fig. 4:**
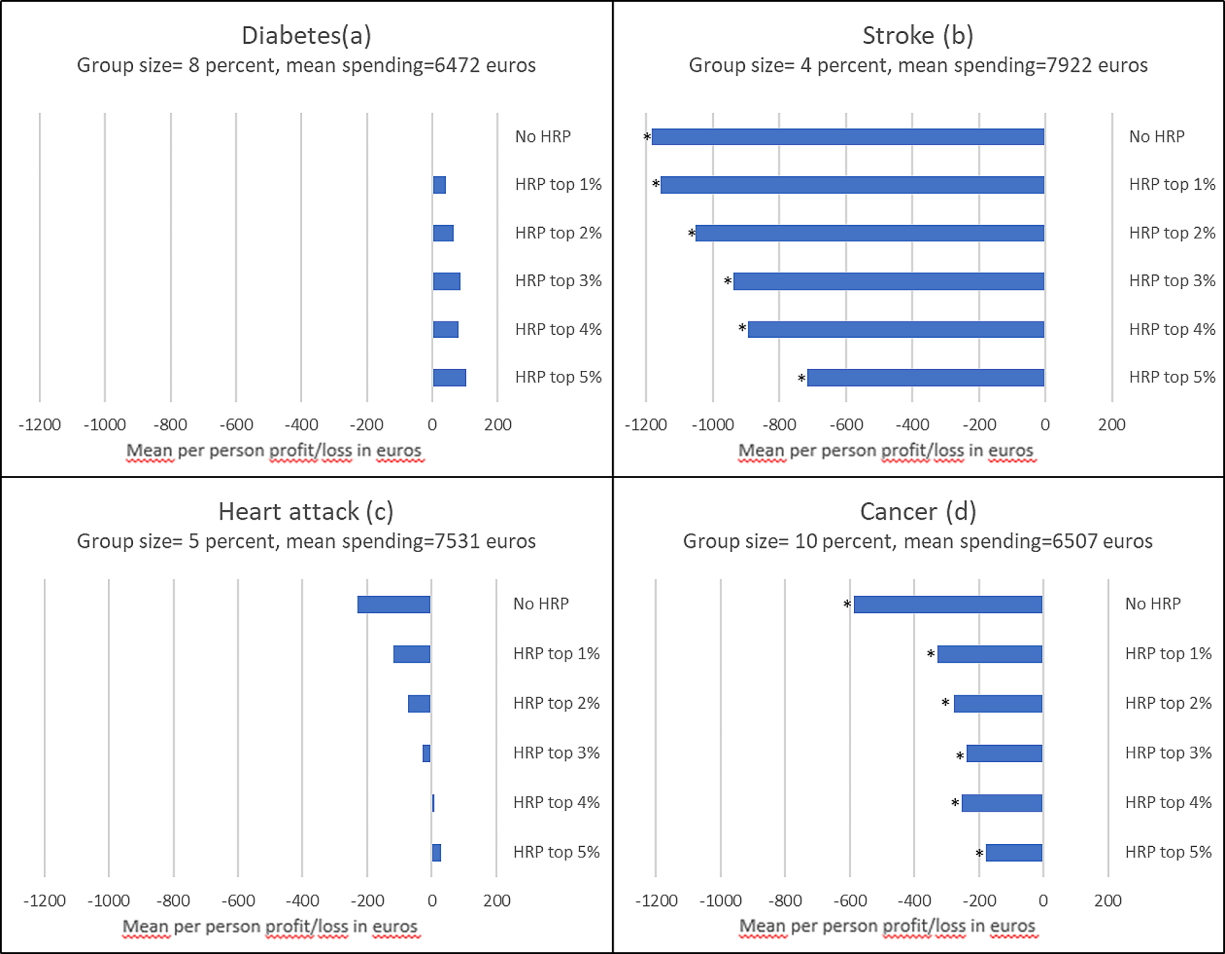
Mean per person profit/loss per year in euros under the Dutch risk-equalization model of 2016 without a high-risk pool and for 5 high-risk pools that vary in size for the groups of individuals who reported to have ever suffered from diabetes, stroke, heart attack or cancer. *Note*: Results are based on test sample (*N* = 115,473). HRP stands for high-risk pooling. No HRP shows the mean per person profit/loss under the risk-equalization model without a high-risk pool. An asterisk (*) means that the presented value is statistically significantly different from zero (*p* < 0.05)

Figure 4 shows the mean per person profit/loss under the Dutch risk-equalization model with and without HRP for individuals who reported to have ever suffered from diabetes, stroke, heart attack or cancer^10^. For stroke, heart attack and cancer the loss decreases with the pool size. For heart attack, the loss even turns into a profit with HRP for the top-4% and top-5% of predicted residual spending (note that for this condition, the predictable profit/loss under all high-risk pooling modalities are not significantly different from zero). For diabetes, the existing profit increases with the pool size, which is related to the fact that diabetes is already well accounted for in the risk-equalization model and HRP further increases the compensation. The relative increase in profit for diabetes is largest between the scenario without HRP and HRP for the top 1% of predicted residual spending (note, however, that the profit for diabetes for these two scenarios is not statistically significantly different from zero). For the groups heart attack and cancer, the relative decrease in loss is again largest between no HRP and HRP for the top 1% of predicted residual spending (48 and 44%, respectively) and decreases further for both groups as the pool size increases. Lastly, for stroke, the pattern of the results differs from the other conditions: the decrease in loss reduces gradually as the pool size goes up.

Appendix A (table A.1) presents the mean per person profit/loss under the Dutch risk-equalization model with and without HRP for nineteen specific conditions individuals reported to have suffered from in the past 12 months. For certain conditions like heart attack, heart condition, cancer and a condition of the blood vessels, HRP strongly reduces the mean per person loss (and sometimes even turns it into a profit, like for heart condition under HRP for the top-3% to top-5%). For most groups, the largest reduction in loss can again be found when moving from no HRP to HRP for the top 1% of predicted residual spending. The results in table A.1 also show that for some condition groups, like migraine, HRP hardly affects the mean per person predictable losses. The reason for this is that individuals with these conditions are most likely not assigned to the high-risk pool.

**Fig. 5:**
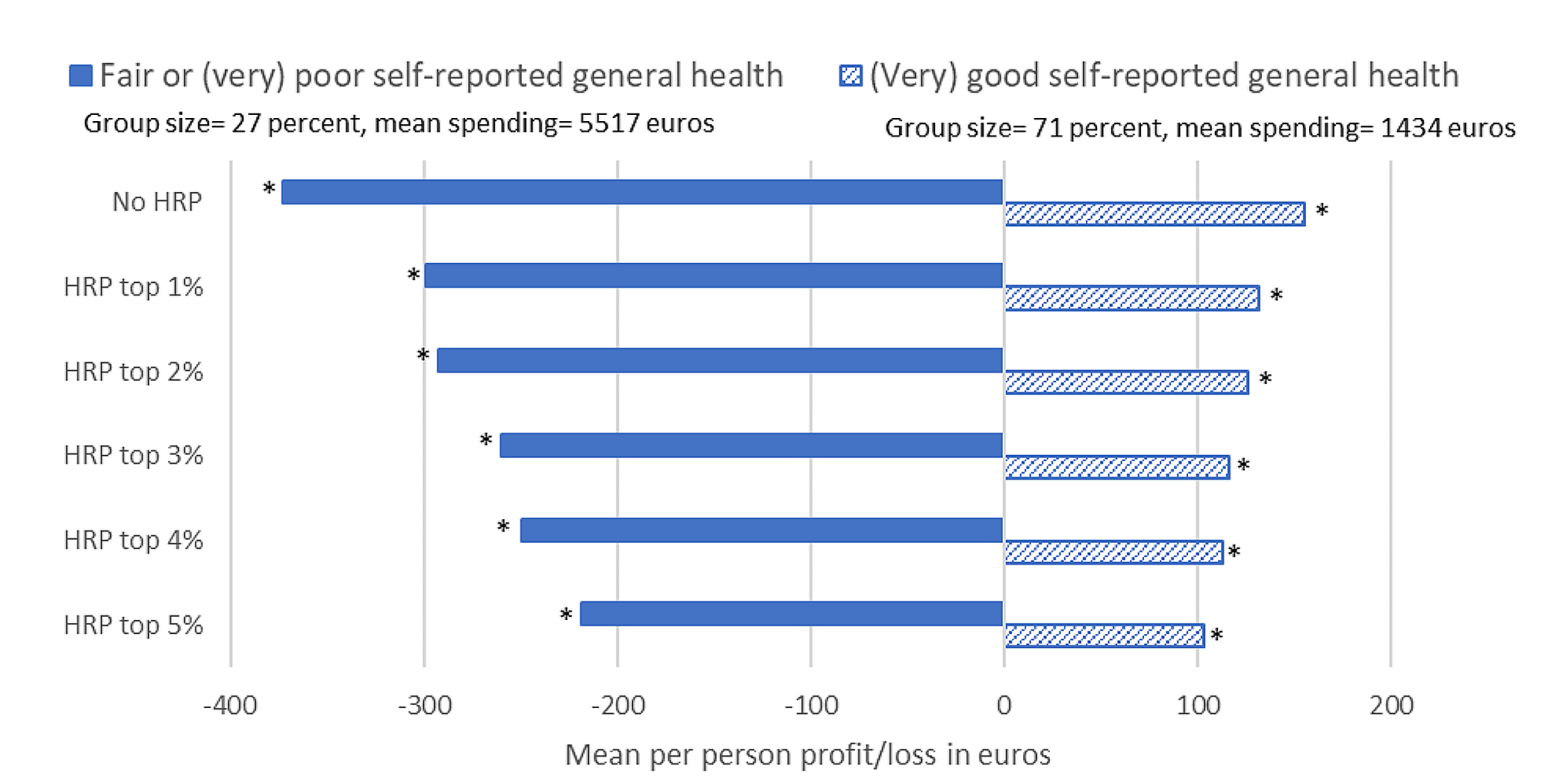
Mean per person profit/loss per year in euros under the Dutch risk-equalization model of 2016 without a high-risk pool and for 5 high-risk pools for the groups of individuals who reported a (very) good general health or a fair or (very) poor general health. *Note*: Results are based on test sample (*N* = 115,473). HRP stands for high-risk pooling. No HRP shows the mean per person profit/loss under the risk-equalization model without a high-risk pool. An asterisk (*) means that the presented value is statistically significantly different from zero (*p* < 0.05). Prevalence of groups do not add up to 100% due to missing values for these specific groups

We also examined the impact of HRP on the profit/loss for groups based on a more subjective measure of health, i.e., self-reported general health. Figure 5 shows the profit/loss for the group who in the prior year reported a fair or (very) poor general health and for the group who reported a (very) good general health. The overall conclusions are similar to those for Fig. 3: both the profit and loss reduce with the increase in pool size. Again, the largest change in profit/loss can be found when moving from no HRP to HRP for the top-1% of predicted residual spending: a reduction of 15% for (very) good general health and a reduction of 19% for fair or (very) poor general health. The absolute difference between these two groups reduces from 530 euros for no HRP to 320 euros for HRP for the top 5% of predicted residual spending (i.e., -40%).

### Incentives for risk selection versus incentives for cost control

The previous section has shown that HRP can strongly reduce predictable profits/losses for specific subgroups, thereby mitigating the incentives for insurers to engage in risk selection. As described, however, like any other form of risk sharing HRP also reduces the incentives for cost control. This section examines the tradeoff between incentives for cost control and risk selection under our five high-risk pool sizes. To indicate the effects of HRP on the incentives for cost control, we simulated the “Power” of the HRP- modalities (see Sect. 3.3.4). The Power-measure ranges from 0 (i.e., no incentives for cost control as payments are based fully on actual spending) to 1 (i.e., full incentives for cost control as payments are completely independent of actual spending). Specifically, we simulated the change in Power for each of the high-risk pool sizes relative to no HRP.

**Fig. 6:**
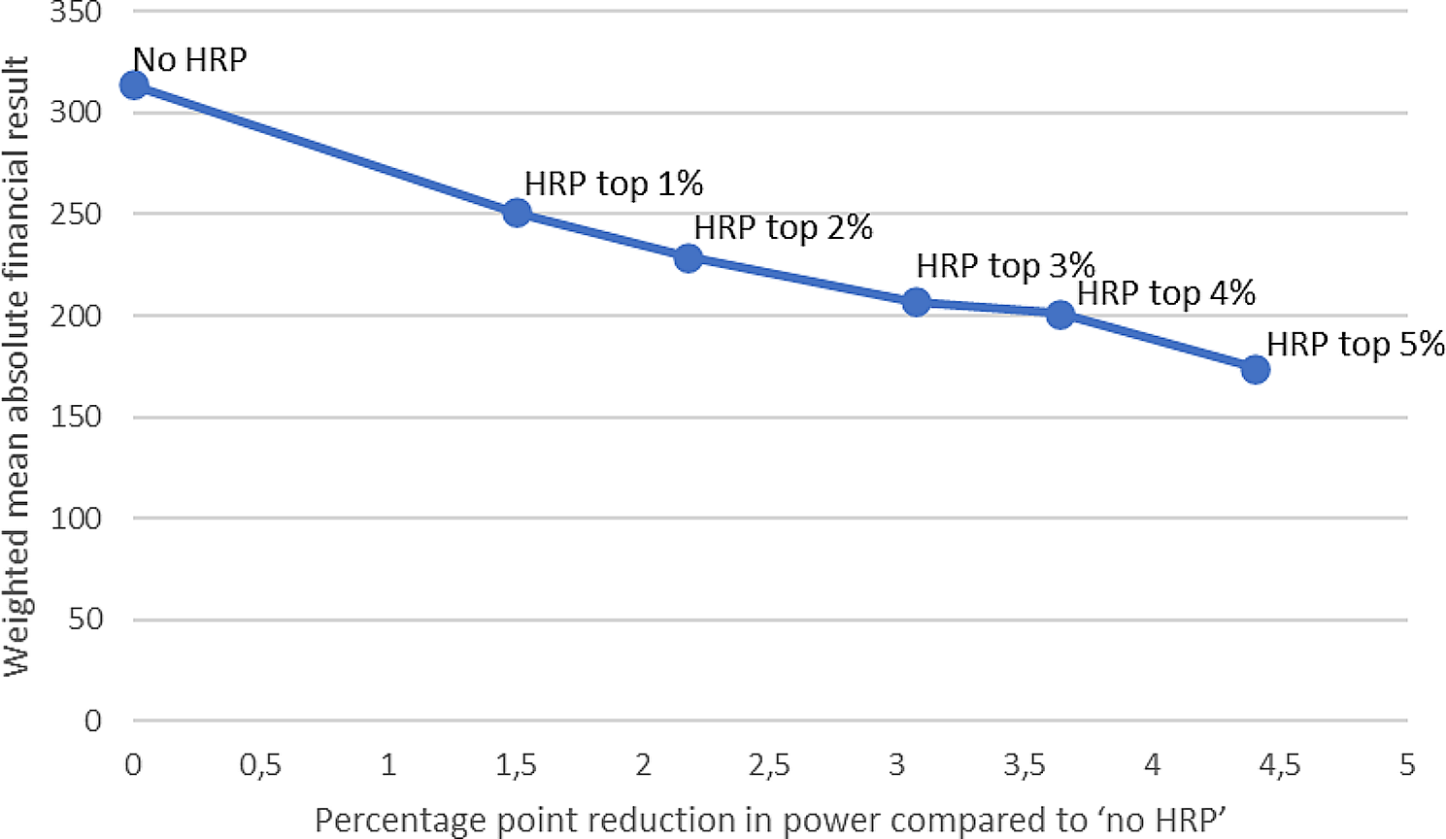
Risk selection incentives (WMAR) plotted against the percentage point reduction in incentives for cost control (Power). *Note*: HRP stands for high-risk pooling. No HRP refers to the situation of risk equalization without a high-risk pool. For the calculation of the weighted mean absolute result (WMAR) the groups no self-reported chronic condition and the groups individuals reported to have ever suffered from diabetes, stroke, heart attack or cancer have been included. See Sect. 3.3.4 for the formulas of the Power-measure and WMAR

Figure 6 plots the percentage point reduction in Power compared to no HRP for the different HRP modalities against the weighted mean absolute result (WMAR); a higher WMAR indicates stronger risk selection incentives. For the WMAR in Fig. 6 we included the subgroup of individuals with no self-reported chronic condition and the 4 subgroups of individuals who reported to have ever suffered from diabetes, stroke, heart attack or cancer respectively (see Sect. 3.3.4).

Figure 6 shows that, the incentives for both cost control and risk selection decrease with the pool size. The largest relative decrease in selection incentives is achieved under HRP for the top 1% of predicted residual spending (decrease of 16%). This comes at the cost of a 1.5 percentagepoint decrease in incentives for cost control (relative to no HRP). The risk selection incentives continue to decrease as the pool size increases, eventually resulting in a total decrease in incentives for risk selection (relative to no HRP) of 43% under HRP for the top 5% of predicted residual spending. Under this modality, the total decrease in incentives for costs control is 4.4 percentagepoints.

To examine the sensitivity of WMAR to the selection of subgroups used, we also calculated the WMAR using other selections of subgroups, namely (1) all subgroups analyzed in this paper, (2) yes/no at least one chronic condition and (3) self-reported general health. Depending on the selection, the reduction in WMAR (i.e., in risk selection incentives) is similar to the results in Fig. 6 and ranges between 33 and 45% under HRP for the top 5% of predicted residual spending relative to no HRP (results not shown). To examine the sensitivity of WMAR to the selection of subgroups used, we also calculated the WMAR using other selections of subgroups, namely (1) all subgroups analyzed in this paper, (2) yes/no at least one chronic condition and (3) self-reported general health. Depending on the selection, the reduction in WMAR (i.e., in risk selection incentives) is similar to the results in Fig. 6 and ranges between 33 and 45% under HRP for the top 5% of predicted residual spending relative to no HRP (results not shown).

## Discussion

In this paper we investigated the potential effects of High-Risk Pooling (HRP) as a supplement to sophisticated risk equalization. A crucial feature of HRP is that it allows for ex-ante assignment of high-risk individuals to the pool, which in our case is done by insurers based on health survey information. In a simulation on data from the Netherlands we examined the effects of HRP on risk selection incentives and incentives for cost control. In a first step, we identified candidates for the pool using a RF model predicting residual spending post risk equalization. We compared identification of high-risk individuals by RF to identification by OLS and found that RF was better able to identify these high-risk individuals than OLS. In a next step, we applied five different pool sizes (i.e., the top-1%, top-2%, top-3%, top-4% and top-5% of the distribution of predicted residual spending respectively) and calculated the mean per person profit/loss for specific subgroups identifiable in the health survey data and compared the results to the situation without HRP. In addition, for each of the pool sizes we evaluated the reduction in incentives for cost control. Our findings suggest that our HRP modality can lead to a considerable reduction in remaining selection incentives by sacrificing a relatively low share of incentives for cost control.

In our simulations, selection incentives (i.e., predictable profits and losses) gradually reduce with a larger pool size. We found the largest marginal reduction in selection incentives when moving from no high-risk pool to HRP for the top 1% individuals with the highest predicted residual spending. When moving from HRP for the top 1% to larger pool sizes (up to the top 5%) the marginal reduction in selection incentives becomes smaller. The reason for this is that our prediction model identifies a more selective group in terms of unprofitable individuals in the 100th percentile of predicted residual spending compared to the 96th to 99th percentiles.

Another finding is that for some chronic conditions HRP reduces profits/losses substantially (e.g., those who suffered from cancer) while for other groups the effect is moderate (e.g., those who ever suffered a stroke) or absent (e.g., those who suffered from migraine in the past 12 months). The reason for this is to be found in the underlying prediction model. The RF procedure only selected the conditions relevant for identifying individuals with high residual spending to include as indicators in the model. Therefore, conditions that are included in the prediction model will be better represented in the top 5% of predicted residual spending and thus benefit more from HRP than conditions that are not included in the prediction model.

As a form of risk sharing, HRP has a direct negative effect on the incentives for insurers to control costs [15, 17, 18]. When regulators only consider this direct effect (as measured by the Power-metric), they are faced with an inherent tradeoff between risk selection and cost control. Depending on how regulators weigh the relative importance of ‘risk selection’ and ‘cost control’, the reduction in selection incentives could outweigh the reduction in incentives for cost control. Although our study ignored such normative weighing, our results do suggest that a relatively small decrease in incentives for cost control can go a long way in reducing remaining selection incentives.

In addition to the direct negative effect, however, HRP might also have indirect positive effects on incentives for cost control. For example, a reduction of selection incentives reduces the expected returns on investments in risk selection, which might lead insurers to turn their attention to other strategies to increase their revenue, such as increasing the efficiency of care. More generally, a reduction of risk selection incentives is expected to increase efficiency as an outcome of competitive health insurance markets [33]. Depending on how the direct and indirect effects play out, the net effect of HRP on cost control may not be negative. However, the indirect effects of reduced selection on efficiency are hard to measure. We believe this is an important direction for further research.

A key assumption underlying this study is that health insurers have additional individual-level information to predict spending that is not included in the risk-equalization model itself. In addition to health survey information, which depending on the context might be challenging to acquire [13], other potential data sources that might be used for HRP are administrative data, multiyear diagnostic data, and diagnostic information from general practitioners [34, 35]. Like the survey information used in this study, these other types of data are also expected to have predictive power regarding residual spending. We do not expect, however, that these other data sources are perfect substitutes for health survey information since the latter includes subjective expectations about future healthcare spending that cannot be picked up (fully) by administrative and diagnostic data. Instead, we expect that health survey information and these other data sources might be complementary when it comes to the assignment of high-risk individuals to the pool. The effects of using these different data sources together for the purpose of HRP remains an empirical question. In addition to alternative data sources, insurers might also use other machine learning techniques, like penalized regression or a super learner [29], depending on the available data. Combined, using more data or other machine learning techniques to identify high-risk individuals might lead to the identification of a more selective group, which when used for HRP might result in a more favorable tradeoff between selection and cost control (given an equal reduction in incentives for cost control). More research is needed to evaluate these options.

To our knowledge, this is the first study to investigate how health survey information can be used to prospectively assign enrollees to a high-risk pool in the context of sophisticated risk equalization. Another strength of this study is that we had access to rich administrative data for all Dutch citizens (*N* ≈ 16.9 m) that we could combine with unique data from a large health survey (*N* ≈ 384k). Also, this study contributes to the discussion of using machine learning techniques in risk equalization. Nevertheless, our study comes with at least two limitations. Firstly, our findings based on the health survey data are conditional on the adult Dutch population of 19 years and older. It is possible that the incentive effects of HRP would be different when considering the total population. Secondly, at least to some extent, the results of this study are dependent on the metrics used. For example, in our simulations of incentives for cost control using the Power metric, we did not include the potential effect of risk adjusters included in the risk-equalization model. Specifically, since the Dutch risk-equalization model includes risk adjusters based on prior year spending (e.g., MYHCG), incentives for cost control are not 100% (i.e., Power < 1) without HRP [32].

Although the outcomes of the HRP design tested in this paper are promising, a practical implementation requires more work. Firstly, HRP comes with several design options which can be chosen such to best fit the specific context in which it is applied. The extent to which alternative designs affect incentives and which design choices yield the optimal tradeoff between selection and cost control is an empirical question and requires further research. As this combination of design choices crucially depends on the weight attached to risk selection and cost control by a regulator, it is important that regulators specify these weights. Secondly, to protect the level-playing field between health insurers, a regulator might need to regulate the administration of the health survey or even supply health insurers with the (results of the) same prediction model to assign individuals to the pool. The reason is that not all health insurers may have the capacity to develop an equally performing prediction model, for instance because of a small portfolio.

Relative to many other possible forms of risk sharing, including proportional risk sharing, reinsurance, and risk corridors [15], an important advantage of HRP is that risk sharing funds are explicitly targeted at *predictably* unprofitable groups, which is an important property in the light of the selection-cost control tradeoff; other forms of risk sharing also compensate for *unpredictably* unprofitable groups, leading to a less favorable selection-cost control tradeoff [19, 15]. In addition, HRP enables health insurers to use information predictive of (residual) health care spending that is not used in risk equalization to improve compensation, which they otherwise might use for risk selection purposes. In that light, we believe HRP is a promising option to mitigate risk selection incentives under sophisticated but imperfect risk equalization.

### Electronic supplementary material

Below is the link to the electronic supplementary material.

Supplementary Material 1

## Data Availability

The datasets generated and/or analyzed during the current study are not publicly available. These data were used under license from the Dutch Ministry of Health, the Dutch Association of Health Insurers and Statistics Netherlands. There are strict guidelines to acquire these data and if people are interested they have to get permission from the third-parties themselves.

## Footnotes

1 We assume that the regulator considers all covered benefits for HRP. Of course, a regulator can also choose to only consider specific benefits for HRP [3]. However, in contrast to including all benefits, only including specific benefits might leave some high-risk individuals unqualified for the pool. Also, not including all benefits could lead to unwanted substitution of health care or gaming [3].

2 Morbidity is defined as at least one classification in a PCG, DCG, MHCG or a DMECG.

3 Because the risk equalization model is estimated by OLS, mean spending equals mean predicted spending in the population. This is not necessarily the case for subsamples of the population, like the health survey sample. The raking procedure already corrected for some of this difference between mean spending and mean predicted spending. To set mean spending equal to mean predicted spending after raking, we multiplied the actual spending with a factor of 1.002457.

4 To acquire the individual-level residual spending from the perspective of a health insurer, we followed a pragmatic approach by re-estimating the actual risk equalization model used in practice. Since a Dutch health insurer has no access to certain socioeconomic information, this replica of the risk equalization model contains the following risk adjusters: age interacted with gender, region, PCGs, DCGs, MHCGs, DMECGs, physiotherapy-spending in the prior year, geriatric rehabilitation care spending in the prior year, yes/no morbidity interacted with age and home care spending in the prior year. We estimated this model on the administrative data containing health care spending for all Dutch citizens. We then calculated the replicated individual-level residual spending by subtracting the spending predicted by the health insurer from actual spending.

5 The optimal number of trees in a RF analysis is when the error rate does not decline any further by adding more trees. In our analyses this was the case at approximately 100 trees, which we found through trial and error.

6 Note that the subgroups were determined on information from year t-1, while the spending, predicted spending and predictable profits/losses pertain to year t. This is also the procedure followed for risk equalization itself, where the information for the morbidity-based risk adjusters is also from year t-1.

7 Note that the percentage increase in spending level per se is irrelevant as the power metric quantifies the ratio between revenue and spending.

8 While the Dutch risk equalization model of 2016 is not supplemented with any risk sharing scheme, the model does include a risk adjuster based on spending levels in prior years which reduces the incentives for cost control [32].

9 The mean residual spending over all these groups (including the group with missing values) is not equal to zero, despite the zero-sum principle of the payment system. This is because the test sample has a mean overall residual spending of -15 euros, which is due to the inherent randomness involved in our split-sample procedure.

10 The mean per person profits/losses for subgroups presented in this section differ from the figures presented in the introduction of this paper based on Van Kleef et al. [4]. Even though the same health survey data are used, the results presented here are based on a selection of the health survey data (i.e., the test sample) while Van Kleef et al. [4] used the total sample.
