# Supplementary material for: High-risk pooling for mitigating risk selection incentives in health insurance markets with sophisticated risk equalization: an application based on health survey information
Source: BMC Health Serv Res. 2024 Mar 4;24:273. doi: 10.1186/s12913-024-10774-x (PMC10913588; doi:10.1186/s12913-024-10774-x)
Supplement: Supplementary file 1 — Supplementary Material 1 [file 12913_2024_10774_MOESM1_ESM.docx]

Appendix A. Mean spending, mean predicted spending and mean per person profit/loss for selective groups identifiable in the health survey under the Dutch risk-equalization model and under five different HRP modalities

*Table A.1 Mean spending, mean predicted spending and mean financial result for selective subgroups identifiable in the health survey under the Dutch risk-equalization model and under five different HRP modalities*

| Groups | Group size | Mean spending | Financial result under different HRP modalities | | | | | |
| --- | --- | --- | --- | --- | --- | --- | --- | --- |
|  |  |  | No high-risk pool | Top 1% | Top 2% | Top 3% | Top 4% | Top 5% |
| Stroke | 1% | 9895 | -1276* | -1212* | -1144* | -1022* | -971* | -973* |
| Heart attack | 1% | 9312 | -1073 | -720 | -706 | -621 | -373 | -358 |
| Heart condition | 3% | 8369 | -374 | -270 | -203 | 50 | 98 | 150 |
| Cancer | 3% | 10419 | -1161* | -158 | -161 | -19 | -125 | -68 |
| Migraine | 12% | 2380 | -113* | -121* | -124* | -114* | -114* | -120* |
| Blood pressure | 22% | 4372 | -103* | -96 | -72 | -32 | -32 | -23 |
| Blood vessels | 4% | 8264 | -1233* | -1131* | -942* | -714* | -717* | -660* |
| Asthma | 9% | 4725 | -198* | -176* | -157 | -110 | -87 | -79 |
| Psoriasis | 3% | 3627 | -282 | -293* | -256 | -256 | -235 | -225 |
| Eczema | 4% | 2832 | -73 | -91 | -90 | -92 | -98 | -92 |
| Severe/recurring dizziness | 4% | 6128 | -755* | -676* | -682* | -655* | -616* | -615* |
| Severe/recurring disease of intestines | 4% | 5334 | -672* | -658* | -645* | -588* | -569* | -565* |
| Incontinence | 8% | 5814 | -236* | -198* | -188* | -150 | -148 | -146 |
| Wear of joint | 19% | 4855 | -295* | -277* | -265* | -261* | -259* | -253* |
| Joint inflammation | 6% | 5818 | -320* | -298* | -301* | -285* | -265* | -263* |
| Severe/recurring condition of back | 11% | 4135 | -208* | -182* | -184* | -178* | -183* | -178* |
| Severe/recurring condition of neck | 10% | 3871 | -219* | -184* | -189* | -189* | -187* | -186* |
| Severe/recurring condition of elbow | 7% | 4531 | -98 | -83 | -62 | -44 | -32 | -33 |
| Other chronic condition | 15% | 4948 | -347* | -262* | -259* | -220* | -202* | -192* |

*Note: An asterisk (*) indicates if the mean residual spending is statistically significant different from zero (p<0.05)*
